# DNA fingerprinting in anthropological genetics: past, present, future

**DOI:** 10.1186/2041-2223-4-23

**Published:** 2013-11-18

**Authors:** Michael H Crawford, Kristine G Beaty

**Affiliations:** 1 Laboratory of Biological Anthropology, Department of Anthropology, University of Kansas, 1415 Jayhawk Blvd., 622 Fraser Hall, Lawrence KS66045, USA

**Keywords:** Anthropological genetics, DNA fingerprints, mtDNA, Variable-number tandem repeats, Y chromosomal and autosomal short tandem repeats

## Abstract

In 1985, Sir Alec Jeffreys developed the variable-number tandem repeat method used to identify individuals and giving researchers the first DNA fingerprints. These initial methods were used in anthropological genetics, a field that uses a comparative approach to answer questions about human history, including the discernment of the origin of Native American populations and the discrimination of clan affiliation from individuals in Siberia. The technological and methodological advances since this time have led to the use of many more markers, including restriction fragment length polymorphisms, Y chromosomal and autosomal short tandem repeats, single nucleotide polymorphisms, and direct sequencing not only to identify individuals, but to examine frequencies and distributions of markers (or “prints”) of entire populations. In the field of anthropological genetics these markers have been used to reconstruct evolutionary history and answer questions concerning human origins and diaspora, migration, and the effects of admixture and adaptation to different environments, as well as susceptibility and resistance to disease. This review discusses the evolution of DNA markers since their application by Sir Alec Jeffreys and their applications in anthropological genetics.

## Introduction

Anthropological genetics is a synthetic field that examines evolutionary theory of interest to anthropologists while applying genetic methodologies [1]. This intimate relationship between genetics and anthropology was first characterized in 1973, in a volume entitled *Methods and Theories of Anthropological Genetics*[2]. This initial synthesis was followed by three volumes on *Current Developments in Anthropological Genetics*[3-5]. The far-reaching impact of the molecular revolution on the field of anthropological genetics in the 1980s and 1990s was assessed by a volume entitled *Anthropological Genetics: Theory, Methods and Applications*[6]. The field of anthropological genetics utilizes a comparative approach on small, isolated populations and topics such as human variation, evolutionary theory, reconstruction of the human diaspora (out-of-Africa), genetic epidemiology, and forensic sciences [7]. Anthropological geneticists (particularly from the Department of Genetics, Texas Biomedical Research Institute) have been successful in mapping quantitative trait loci involved in biological pathways of diseases such as diabetes mellitus, cancers, obesity, osteoporosis, and coronary heart disease [8]. Schanfield has reviewed the prominent role of anthropological genetics in cases of legal interest, using classic genetic markers and molecular methods [9]. See the thematic review of the application of DNA fingerprints to forensic sciences in this special issue of *Investigative Genetics*. In population studies, genetic markers have been defined as “discrete, segregating genetic traits which can be used to characterize populations by virtue of their presence, absence, or high frequency in some populations and low frequencies in others” [10]; in a sense, a combination of these markers can be used as a “fingerprint” of a population. Although this definition was first applied to blood groups and protein variation, any segregating regions of DNA, present in some populations but absent or infrequent in others, may be termed genetic markers. Thus, variable-number tandem repeats (VNTRs), short tandem repeats (STRs), mitochondrial DNA haplogroups, Y-specific non-recombining region (NRY) haplotypes, and single nucleotide polymorphisms (SNPs) have been used as “genetic markers” to document population history and to assess the actions of the forces of evolution. This thematic review focuses on the application of a variety of genetic markers (from VNTRs to STRs to SNPs) to the resolution of several evolutionary controversies. Examples of the application of these DNA fingerprints (genetic markers) to evolutionary questions come primarily from studies conducted by researchers of the Laboratory of Biological Anthropology at the University of Kansas, and provides a more “personalized view” of anthropological genetics that has built upon the work that Sir Alec Jeffrey began over 35 years ago.

## Review and discussion

### DNA fingerprints

In 1985, Alec Jeffreys and his colleagues developed a method using VNTRs or minisatellites of DNA to identify specific individuals for forensic purposes and parenthood determination [11]. These DNA fingerprints are specific to an individual (or to a set of monozygotic twins) with 1 in 30 billion chances that the identical patterns will be encountered in an unrelated individual. Southern blot methodology was utilized to identify specific loci and alleles from a multitude of DNA fragments. This method involved cutting intact DNA with a sequence specific restriction enzyme, followed by separation of fragments using electrophoresis, transferring these fragments onto a nitrocellulose membrane, and hybridizing the fragments with specific probes labeled by radioactive isotopes or biotin. Numerous minisatellite loci were considered simultaneously, which increased the observable variation but made it difficult to discern specific alleles. A series of fragments of various lengths were digitized and grouped into size bins and the frequencies of fragments within these bins were calculated for each population. Because of the time-consuming nature of this methodology and the ambiguity associated with whether fragments within bins were specific alleles, this Southern blot method was eventually supplanted by PCR-based assays [12]. The PCR methodology is less expensive, more sensitive, less time consuming and amplifies the specific regions of DNA, using multiplexes and “cocktails” containing thermostable DNA polymerase.

### Anthropological genetic applications of DNA fingerprints

#### The past

**Figure 1:**
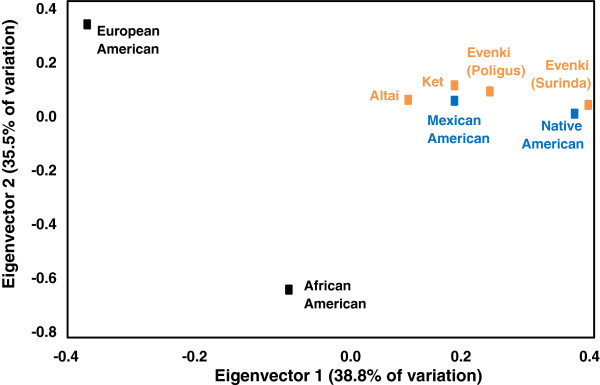
**Least square reduction of an R-matrix plot based on allelic frequencies from 5 VNTR loci (*D7S104*, *D11S129*, *D18S17*, *D20S15*, and *D21S112*).** Figure adapted from McComb et al. [17].

During the late 1980s and early 1990s, frequency distributions of VNTRs were used as genetic markers to discriminate between ethnically defined populations [13-15]. In addition, because of the non-coding nature of VNTRs, high mutation rates, and high genetic diversity, McComb et al. applied VNTR restriction fragment length polymorphism distributions to questions concerning the peopling of the Americas and the characterization of the genetic structure of indigenous Siberian populations [16-18]. Data assessing morphological traits and classic genetic markers suggested a Siberian origin of Native American populations, but until 1989, DNA samples from Siberian indigenous groups were not available to western scientists to verify this origin. Field investigations in Siberia were made possible by the breakup of the Soviet Union and “perestroika” (rebuilding). During the summers of 1989–1993, an international team of researchers from the University of Kansas and the Russian Academy of Sciences, funded by the NSF, collected blood samples from volunteers in two adjacent Evenki reindeer herding brigades (Surinda and Poligus), a small Ket fishing/hunting village on the Yenesei River (Sulamai), and a cattle-herding village from Gorno-Altai (Mendur-Sokhon). In 2002, DNA samples were collected from Even, Koryak, and Aleut communities of Kamchatka and Bering Island. DNA was extracted at the Laboratory of Biological Anthropology, University of Kansas, and analyzed using Southern blots to assign DNA fragments into length bins through digital comparisons with sizing ladders. All statistical analyses were based on a conservative standard error of ± 2%. Intergroup variation was tested for statistical significance using the Kolmogorov-Smirnov test with Bonferroni correction for multiple comparisons (*P* = 0.05). Siberian populations clustered with the Native American groups were statistically significantly different from European and African Americans [17] (Figure 1). In addition to DNA fingerprints, mtDNA analyses of the same DNA samples demonstrated that Siberian and Native American populations shared the founding haplotypes A, B, C, and D [19]. Phillips-Krawczak et al. later identified the presence of a Siberian X haplogroup in the Kizhi population of Gorno Altai [20]. Non-recombining Y chromosome markers further verified the Siberian origins of Native Americans [21].

Crawford et al. also utilized VNTR loci to determine clan affiliation in the Kizhi pastoral community of Mendur-Sokhon, Gorno Altai region of Southern Siberia [22]. A sample of Altai Kizhi were characterized for three VNTR loci (*D7S104*, *D11S129* and *D18S17*) and linear discriminant function analysis was used to classify unknown individuals to a specific clan. The Kizhi community contained three major clans, Irkit, Todosh, and Kipchak, and other smaller clans. Linear discriminant function correctly classified 72% of all unknowns entered into the analysis. The highest correct classification occurred when 80% of the research subjects were placed in the Todosh clan, followed by 75% correct classification of individuals assigned to the Irkit clan, and 60% into the Kipchak clan. Those Kizhi individuals who were not affiliated with the Irkit, Todosh, or Kipchak were assigned randomly to a fourth group. If all of the clan assignments were random in regards to the VNTR loci, individuals would have been correctly assigned 25% of the time, while the unassigned individuals were classified into that category 29% of the time. These data suggest that VNTR markers have detected genetic similarities within each clan that permit a high probability of correct assignment of each individual to a correct clan (Table 1).

**Table 1:** **Linear discriminant function analyses based on VNTR loci classification of individuals into specific patrilineal clans (Crawford et al.**[22]**)**

**Put into group**	**Irkit**	**Todosh**	**Kipchak**	**Unassigned**
Irkit	9	0	1	9
Todosh	2	8	1	5
Kipchak	1	0	6	8
Unassigned	0	2	2	9
Total number	12	10	10	31
% Correct	75%	80%	60%	29%

### Present

#### Microsatellites (STRs)

Technological advances have allowed for more efficient means of investigating the genetic makeup of individuals with the use of DNA fingerprints such as STRs. In anthropological genetics, these markers have been used as ancestry-informative markers to reconstruct the human diaspora and to interpret the evolutionary history of human populations to answer questions of population origins, migration, and admixture. STRs, also known as microsatellites, are sequences of 2 to 6 base pairs (bp) repeated in a region of DNA from 3 to 100 times. Variant alleles usually result from slipped strand mispairing during DNA replication. In this review, we focus on the anthropological genetic questions that have been investigated during the last decade using STRs. STR variation can be examined in a number of different ways to test hypotheses concerning anthropological genetics. The following examples demonstrate the usefulness of STRs in answering evolutionary questions, such as (1) Are the Basque inhabitants of Spain and France remnants of the Paleolithic populations of Europe prior to the expansion of agriculture and Indo-European languages from the Middle East, circa 10,000 years B.P.? Are they Iberian groups that have been geographically isolated from their neighbors or are they related to distant populations from North Africa or the Caucasus? (2) How much gene flow did the populations of the Aleutian Islands experience from Russian, English, and/or Scandinavian sources? (3) Can a single ubiquitous STR allele (*D9S1120 9 RA*) reveal the number of migrations that have occurred from Siberia into the Americas?

### STRs and Basque origins

Are the Basque populations remnants of the Paleolithic settlers of Europe and/or do they show affinities to populations of the Caucasus or North Africa? Most of the early molecular genetic studies of Basque populations were based primarily on small samples of school children or adults from urban sites, with some admixture with the surrounding Spanish communities [23]. The Vizcaya Province sample (68 unrelated volunteers) revealed, on the basis of 13 autosomal STR loci, that the Basques are outliers relative to neighboring Spanish and the more distant North African populations. Young et al. characterized a total of 404 DNA samples for nine autosomal STR loci collected from rural villages and towns of four Basque Provinces [24]. Multidimensional scaling based on Shriver’s D_sw_ distance matrix did not support the hypothesis of a recent common ancestry between the Basques and populations from the Caucasus or North Africa [25]. STR, mtDNA, and NRY genetic markers indicate that the Basques are distinct from the surrounding Spanish populations but also differ from the inhabitants of the Caucasus and North Africa. The most parsimonious explanation for the distribution of the genetic markers is that the contemporary Basques are descendants of the earliest Paleolithic migrants into Europe. However, recent analyses of ancient DNA from early Neolithic farmers and hunter-gatherers suggest that the maternal genetic contribution of farmers coming from the Middle East is higher than previously suspected [26,27].

### Aleutian island admixture

**Figure 2:**
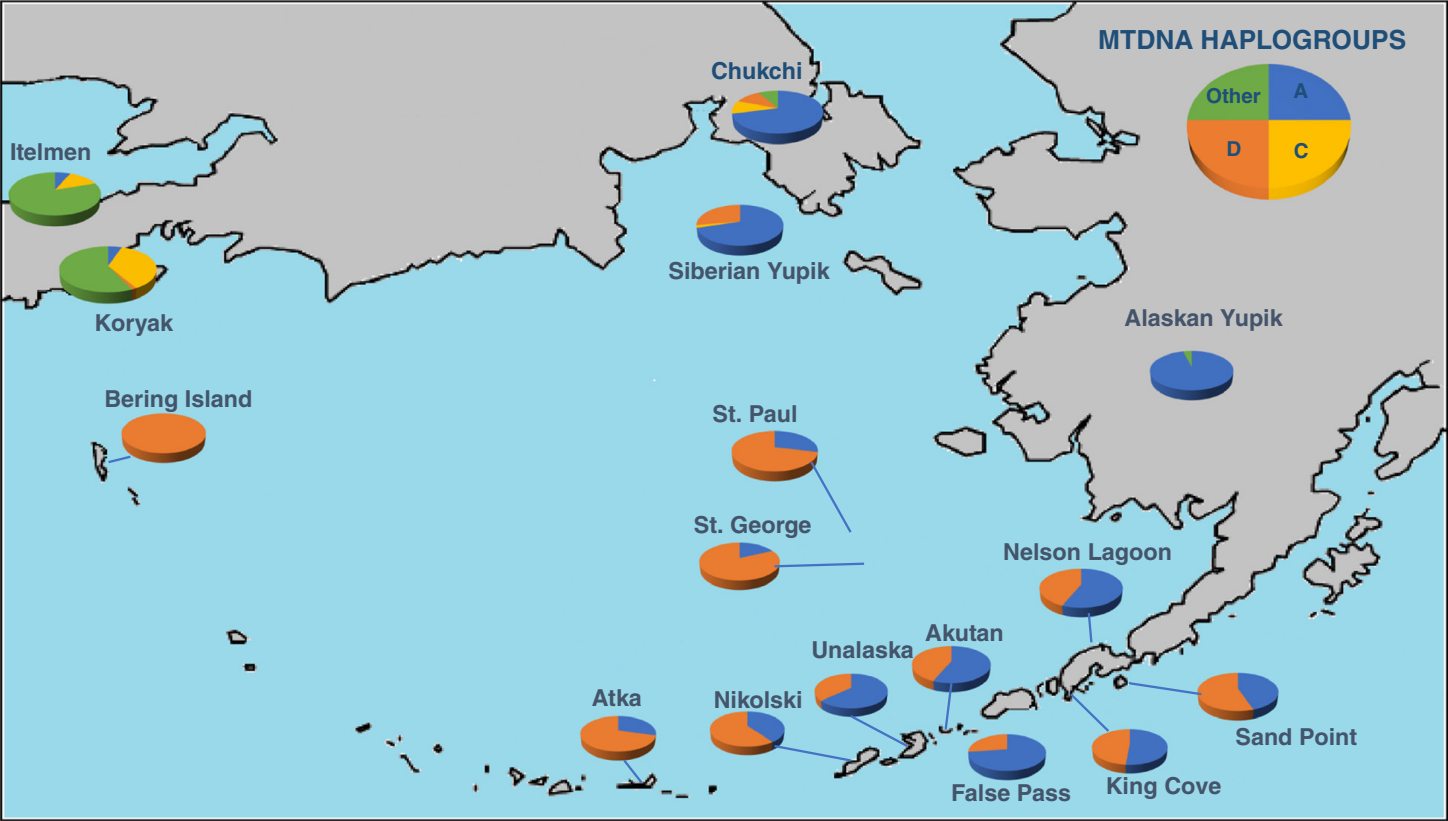
**Frequency of mtDNA haplogroups present in the Aleutian Islands determined by restriction fragment length polymorphisms and hypervariable segment-1 sequences, adapted from Crawford et al.**[28]**.** Only haplgroups A (shown in blue) and D (shown in orange) are present in the Aleutian Islands, whereas haplogroup C (shown in yellow) and other mtDNA haplgroups (shown in green) are found on the Alaskan mainland and Siberia.

**Figure 3:**
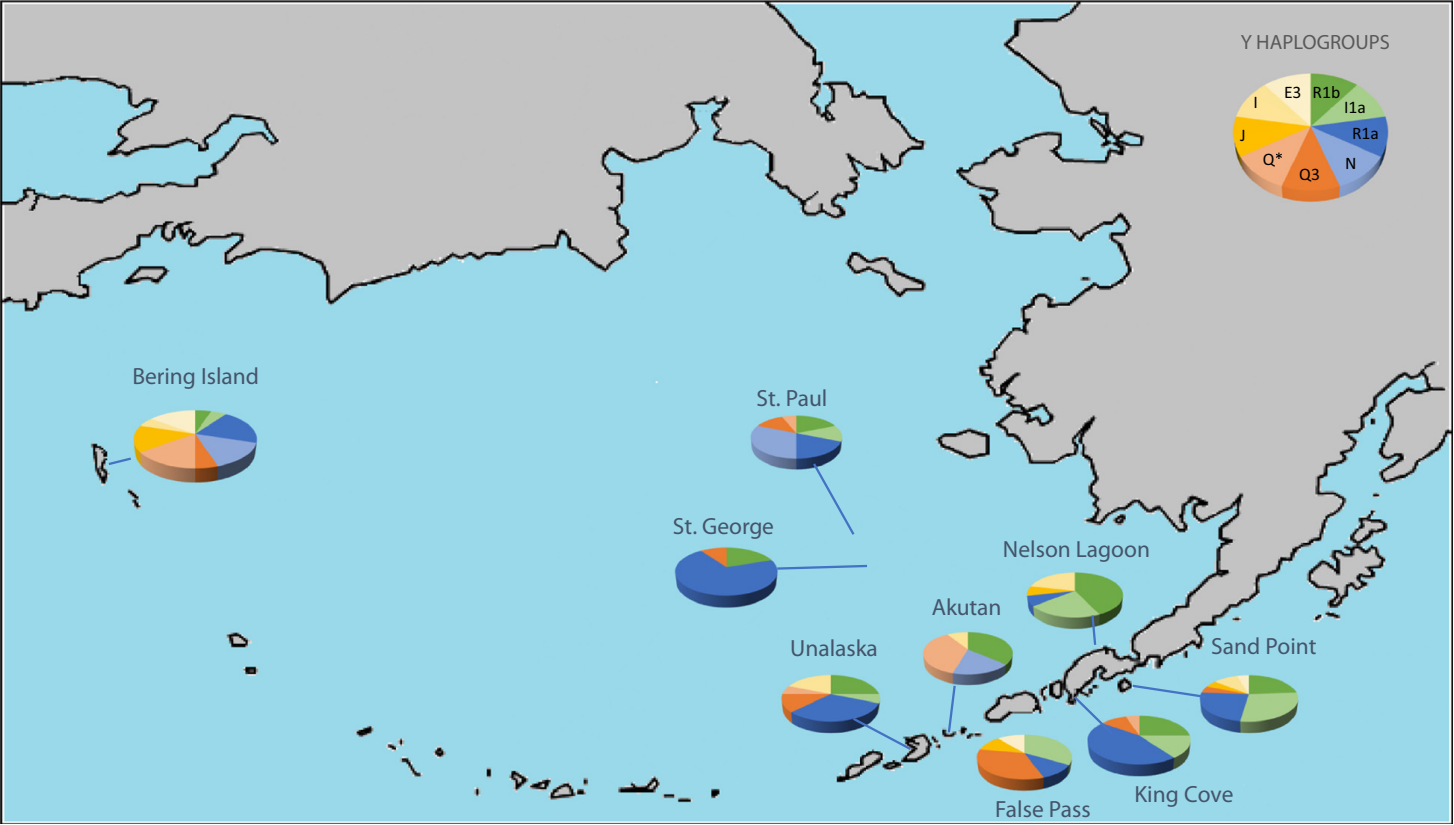
**Frequency of Y haplogroups present in the Aleutian Islands determined by SNPs and STR haplotypes, adapted from Crawford et al. with data from Rubicz et al.**[28,29]**.** Haplogroups shown in green represent haplogroups found in high frequencies in Western Europe, haplogroups shown in blue are found in high frequencies in Russia, and haplogroups in orange are believed to be native to Aleuts.

Estimates of gene flow and admixture in human populations may vary depending on which specific genetic markers are used to characterize the populations. If the indigenous Aleutian island populations are characterized solely by mitochondrial DNA haplogroups shown in Figure 2, only the native haplogroups A (shown in blue) and D (shown in orange) are observed [28]. Based solely on these data, one might conclude that there was no gene flow from Russian, English, or Scandinavian populations into the Aleutian Islands. However, morphologically, the Aleuts appear to be highly admixed. In Figure 3, NRY haplotypes based on SNPs indicate that only 15% of the Y chromosomes from male participants of the Aleutian archipelago were either Q* or Q3 (shown in light orange and orange), considered Native American paternal lineages [29]. Thus, 85% of the Y chromosomes of the Aleutian Islands inhabitants are of European origin, primarily R1b (dark green) or R1a (dark blue), depending on whether the samples are from the western or eastern islands [30]. The calculation of admixture (using the program Admix 3.1) based on nine autosomal STR loci revealed that approximately 40% of the genes in the Bering gene pool were of Russian origin while 60% were Aleut. Genetic markers that recombine, such as STRs, provide a more accurate assessment of the total contents of an admixed gene pool in human populations, but fail to detect gender-specific patterns of gene flow.

### Private STR alleles and migration into the Americas

**Figure 4:**
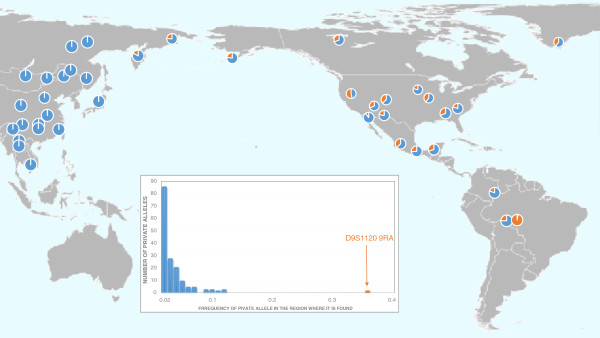
**Distribution of the *D9S1120 9 RA *allele shown in orange.** Redrawn following Schroeder et al. [32].

The frequencies of private STR alleles and their ubiquitous distributions can provide invaluable information concerning the evolutionary history of populations. Schroeder et al. described a private STR allele (*D9S1120 9 RA*), which is ubiquitous in the Americas but present in only two indigenous Siberian populations, Koryaks and Chukchi, both groups located proximally to the former location of the land bridge, Beringia (Figure 4) [31]. While this private allele, shown in orange, is frequent in the Americas and in two Siberian populations, it is absent in Europe, Africa, Australia, Oceania, and most of Asia. The most parsimonious explanation for the geographic distribution of this private allele is that an ancestral Siberian population migrated across the Bering land bridge in a single wave. This single migration theory is based on the assumptions that all copies of the 9-bp allele are identical by descent and not influenced by selection. Schroeder et al. tested these underlying assumptions by examining the haplotypic background in the vicinity of *D9S1120*[32]. They observed that 91% of these chromosomes share the same 76.26 kb haplotype that they termed “American Modal Haplotype”. Schroeder et al. suggest that the high frequency and widespread distribution of the 9-repeat alleles are unlikely to be the result of natural selection [32]. They conclude that all contemporary Native Americans and Western Beringians can trace their ancestry to a single founding population.

Recent analyses of genome-wide SNP data suggest multiple waves of migration from Siberia to the Americas [33]. The exact number of migrations is difficult to assess because of the few North American populations analyzed in this sample (n = 4). Reich et al. conclude that three migrations occurred (the same number postulated by Greenberg et al. [34]), consisting of Amerinds (earliest migrants), NaDene (Canada and SW United States), and Aleut/Eskimo (last arrivals) [33]. How can these differing conclusions be reconciled? One possible scenario is that multiple migration waves came from the same Beringian source population. Among Siberian populations, only the Altai share all of the founding mtDNA haplogroups A, B, C, D, and X. Yet, these Central Siberian groups are located more than 1,000 miles from Beringia with numerous genetically distinct populations located between the Altai and the region closest to Beringia, Chukotka. Does the Altai region share common ancestry with the populations that founded the Americas? An alternative explanation is that the multiple migrations were followed by extensive gene flow between the migrant groups, thus, spreading the private allele. A third possible explanation is that the STR mutation occurred on the land bridge, followed by gene flow into both the Americas and Siberia. This ubiquitous mutation is found in all Native populations of the Americas tested to date, but in only two contemporary Siberian groups, Chukchi, located on Chukotka, which is adjacent to Beringia and Koryaks, located south of Chukotka on the Kamchatkan peninsula (Figure 3).

### DNA sequencing and the reconstruction of evolutionary history

In the late 1970s and early 1980s, DNA sequencing, which allows for direct identification of individual or population fingerprints, was a costly and time-consuming methodology inappropriate for use in population genetics due to the required sample size. As a result, most of the early sequencing in anthropological genetics was focused on hypervariable segment-1 of mtDNA, a non-coding region that contains considerable genetic variation, approximately 400 bp in length. However, vast expanses of genomic DNA were useful for determining the structure and function of specific genes. High throughput DNA sequencing methodologies and machines have made large samples from specific populations economically feasible, with a cost, projected by the National Human Genome Research Center, of $1,000 per genome within the next year [35]. Sequencing human genomes yields large numbers of SNPs that can be considered equivalent to fingerprints or genetic markers.

One application of whole genome sequencing is its application to questions of admixture and gene flow. Measures of admixture and gene flow were initially based on estimates of the frequencies of marker genes in parental populations and compared to frequencies in the admixed groups. Earliest attempts to ascertain the proportions of African and European genes in African Americans depended on frequencies of Rhesus blood group R_o_ in an African American population and compared to estimated frequencies of these alleles in West Africa and Europe [36]. This proportion of admixture (m) was computed using the Bernstein (1931) formula:

(1) $$m=q_{h}–q_{2}/q_{1}–q_{2}$$

where, q_h_ is the frequency of the allele in the admixed population; q_1_ and q_2_ are frequencies of the same allele in the parental populations [37].

In the 1960s and 1970s, Bernstein’s method for estimating admixture for populations with two ancestral groups was expanded for populations with three or more parental groups using parental frequencies and maximum likelihood, true least squares, or multiple regression statistical approaches [38]. All of these approaches suffered from the same weaknesses, i.e., the parental frequencies were rough estimates from populations dating back centuries before.

Halder et al. developed a panel of ancestry informative markers (AIMs) consisting of SNPs for estimating individual bio-geographical ancestry and admixture. These are genetic loci with large frequency differences between ancestral populations allowing them to act as “prints” or marks of a specific population [39]. They initially employed 176 autosomal AIMs from four continents, namely Europeans, West Africans, Indigenous Americans, and East Asians. This approach for estimating admixture based on AIM SNPs was effectively applied to two Mexican American samples from San Antonio, Texas, to determine if their genetic structures were equivalent [40]. A total of 706 participants from the San Antonio Family Diabetes Study (SAFDS) were compared to 586 males from the San Antonio Center for Biomarkers of Risk of Prostate Cancer (SABOR) using 64 ancestry informative markers. Significant genetic differences in population structure were observed in the ancestral proportions of the two samples of Mexican Americans from San Antonio. The SAFDS sample exhibited 50.2 ± 0.6% European admixture, while the SABOR sample had 58.9 ± 0.7%. Similar differences were observed using this method for estimating Native American proportions, SAFDS 46.4 ± 0.6% versus SABOR 38.2 ± 0.7%. The West African admixture was estimated at 3.1 ± 0.2% for the SAFDS sample and 2.9 ± 0.2% for the SABOR Mexican American samples from San Antonio. These AIM (SNP) methodologies are considerably more robust and provide more informative estimates of admixture than standard genetic markers, mtDNA, or NRY haplotypes in subpopulations.

Because of high throughput sequencing and the characterization of entire genomes, Johnson et al. have been able to reconstruct the history of admixed populations using DNA recombination to parse out the more specific geographical sources of the parental populations [41]. The shorter chromosomal segments reflect a longer evolutionary history because they have had more time to recombine with unrelated DNA; the longer chromosomal segments reflect a more recent admixture. By comparing DNA segments from one ancestral population (either European, African, or Native American) with admixed groups, greater accuracy can be obtained about the origin of the parental groups and the sizes of the source of the gene flow. They found that the European contribution to the Latino population came from Spain and Portugal and had a low genetic diversity, indicating that few individuals contributed to the admixed population [42].

Among the projects underway to better understand genome wide diversity is the 1000 Genomes Project, which is currently sequencing 2,500 genomes from individuals from all over the world in an attempt to reveal the extent of the diversity contained in the human species and determine how this genetic diversity translates into specific phenotypes [43]. This project has identified several hundred thousand SNPs that vary in allelic frequencies by population, exposing potential variants that will allow us to better define and reconstruct the human diaspora, provide a better understanding of ancestry at both the individual and population level, and allow us to better tell the story of both ancient and recent admixture. These data will initiate a new era of anthropological genetics and will further shift the definition of what constitutes a genetic marker or DNA fingerprint.

### Ancient DNA (whole genome)

The last decade has also seen an emergence of technology that has allowed for investigation of ancient genomes beyond mtDNA, traditionally a focus in ancient molecular studies because of the abundance of mitochondria in skeletal remains. These advances have included the sequencing of entire genomes of ancient remains of Neandertals and a hominin group from Siberia, called Denisovans, that were identified by their unique genetic characteristics [44,45]. These studies have shown that we shared a common ancestor with Neandertals and Denisovans some 800,000 years ago [45], that Neandertals have contributed more genes to non-African populations than African populations [46], and that Denisovans have contributed to the genomes of Melanesians, Australian aborigines, and Southeast Asians [45]. Studies of both groups of ancient hominins have also unraveled functional genes. For example, Neandertal remains from various sites indicate the presence of type O blood [47], alleles that may be associated with red hair and fair skin [48], and the ability to taste the bitter chemical phenylthiocarbamide [49]. Genetic variants of the Denisovan individual suggest the presence of dark skin, hair, and eyes [45]. These advances have allowed us to look further back into our evolutionary history and allow us to better refine our knowledge of how, when and why we have come to be.

In anthropology, whole genome studies of ancient individuals have also been used to answer questions regarding the peopling of the Americas. A human hair tuft, excavated in 1986 at Qeqertasussuk, a Saqqaq archeological site from West Greenland, was rediscovered in a museum in Copenhagen. Because of the permafrost conditions, there was excellent preservation of both mitochondrial and genomic DNA. The whole mtDNA genome was first sequenced from this Paleo-Eskimo, dating back 4,000 to 5,000 years B.P. [50]. The mtDNA haplogroup (D2a1) detected in this Paleo-Eskimo is distinct from modern Native Americans and Neo-Eskimos but is identical to the haplogroup observed in contemporary Aleuts of the Archipelago [50]. This analysis raised questions about a potential early migration of Siberians who expanded into Greenland prior to the later Thule Eskimo expansion.

Rasmussen et al. sequenced the whole genome of the Paleo-Eskimo and recovered 353,151 high confidence SNPs [51]. This Saqqaq genome clusters with Asian populations instead of the contemporary Eskimo or Native American populations. The maternal discontinuity first described by Gilbert et al. was further verified through whole genomic sequencing [50].

Because of the identification of the vast array of SNPs in the Saqqaq genome, it was possible to identify the functional SNPs in this 4,000 year old Paleo-Eskimo. Rasmussen et al. utilized the observed SNPs to reconstruct the following phenotypes of Saqqaq man: blood group subtype *A1*, Q1 NRY haplogroup, brown eyes, non-European light skin, increased risk of baldness, higher body mass index, dry cerumen, shovel-shaped incisors, and a metabolism that was adapted to a cold environment [51]. These phenotypes were deduced from their associations to SNPs, such as a single base deletion in a transferase gene that results in an additional domain at the carboxyl terminal and an A1 phenotype [52]. Similarly, the presence of a non-synonymous variant (C/C) in the *TP53* on chromosome 17, suggested that Saqqaq man possessed a more active form of *p53* by coding for an *Arg* variant which is related to the more effective regulation of metabolism in cold climates [53]. Similar functional associations may yield future information about the evolution of complex diseases and the genetic predispositions for chronic conditions, such as heart disease or breast cancer, in contemporary and ancient populations.

### Future

With the rapid changes in technology and data analyses, DNA genetic markers will play a significant role in future anthropological genetics. Whole genome sequencing is going to become cheaper and faster. The main hurdle for scientists will be the analysis of immense data sets (millions of nucleotides) that are being generated by massive sequencing programs. Within anthropological genetics, these developments are going to mean improvements in the use of molecular data in forensics (with less reliance on more subjective morphological techniques), genetic epidemiology, and population genetics. Greater emphasis can then be placed on unraveling the cultural and environmental factors that shape the expression of our genomes.

Anthropological geneticists investigating disease associations and adaptation have long worked toward uncovering the genetic variation that leads to disease and disease susceptibility. These attempts have, over the past decade, generally been performed using genome wide association studies that have identified some common variants that can lead to, or provide protection from, pathology. However, many of these diseases and disorders may be caused by rare variants that do not give a strong enough signal for identification (see Gibson, 2012 for a review [54]). The 1000 Genomes Project may rectify some of these shortcomings as it aims to identify variants that are found at a frequency of 1% compared to the frequency of common variants used in genome-wide association studies that are found at roughly 5%. Furthermore, whole genome sequencing will reveal rare variants that lie farther from the block of linkage disequilibrium that may also influence the disease pathway. These data will only expand as more studies involve the use of whole genome sequences towards a better understanding of disease.

Future studies of admixed populations will be based on whole genomic sequencing, the effects of recombination, linkage disequilibrium and the use of panels of ancestry informative markers. In the past, the effects of natural selection on admixture estimates could only be examined using imprecise approaches such as the examination, locus by locus, of deviations from expectation under a specific gene flow model. Through the use of whole genomic sequencing, regions of the genome can be examined for the signature of selection in both modern and ancient populations. In addition, rare alleles found only in specific groups should allow for a more detailed picture of human history and better define the complicated ways in which humans interact with one another and the environment.

In the 1980s, Sir Alec Jeffreys first pioneered DNA fingerprints as a means of identifying individuals. Since that time many more genetic markers and polymorphisms have been developed to identify unknown individuals of forensic interest. Now, an individual’s entire genome can be considered a DNA fingerprint, but its size, and the computational power necessary for analysis, makes its use in forensics inefficient and costly. The changing technology has resulted in the discovery of many more genetic markers (mtDNA, NRY, autosomal STRs, and SNPs) that are better suited for forensic and anthropological analyses, as well as cheaper and faster ways of achieving these analyses.

The future application of genetic markers (DNA fingerprints) is wide open and the next decade of research will lead to a better understanding of the origins and evolution of our species. It is unclear how far back in time studies of ancient DNA will take us, but these new methodologies will provide anthropologists with a refined story of human history, unraveling the complexities of human migration, admixture, and the successful and unsuccessful ways in which hominin genomes were selected by their environment. We are in the initial stages of personalized medicine in which our familial genomic endowment will determine specific treatments. We envisage a future where genetic information, a fingerprint of an individual’s genome, will be readily available and utilized for the assessment of ancestry, health risks and the treatment of disease, and crimes will be solved by comparisons of DNA from individuals of interest in particular cases with huge DNA data bases. When Sir Alec Jeffreys first began his work using fingerprints to identify individuals for forensic purposes, it opened a door to research that has allowed a better understanding of who we are both as individuals and as a species.

## Abbreviations

AIMs: Ancestry informative markers; NRY: Y-specific non-recombining region; SABOR: San Antonio center for biomarkers of risk of prostate cancer; SAFDS: San Antonio family diabetes study; SNPs: Single nucleotide polymorphisms; STRs: Short tandem repeats; VNTRs: Variable-number tandem repeats.

## Competing interests

The authors declare that they have no competing interests.

## Authors’ contributions

MHC and KGB participated in the writing of the manuscript. Both authors read and approved the final manuscript.
